# Hospitalization of patients with schizophrenic and affective disorders in Israel in the aftermath of the structural and rehabilitation reforms

**DOI:** 10.1186/2045-4015-2-29

**Published:** 2013-07-23

**Authors:** Daphna Levinson, Yaacov Lerner

**Affiliations:** 1 Mental Health Services, Ministry of Health, 39 Yermiyahu st, Jerusalem, 9101002, Israel; 2 Falk Institute for Mental Health Research, Kfar Shaul Hospital, Givat Shaul, Jerusalem, 91060, Israel

**Keywords:** Psychiatric hospitalization, Length of stay, Psychiatric reforms

## Abstract

**Background:**

In the last decade (2001–2010) the Ministry of Health implemented two major inter-related reforms: a ’structural reform’ to reduce the number of psychiatric beds and the ’Rehabilitation of the Mentally Disabled in the Community Law’, which allocated funds for a variety of residential and vocational programs in the community for these patients. The objective of the present paper was to examine the impact of the two reforms on the hospitalization of schizophrenic and affective disorder patients by tracking the patterns of their inpatient care during the last decade.

**Methods:**

Data on all psychiatric admissions during the period 1990–2011 were extracted from the Israel Psychiatric Case Register to examine changes in the rate of admissions, length of hospitalizations, total inpatient days and tenure in the community. The analysis was done separately for first-in-life vs. all admissions and for patients with schizophrenia vs. patients with affective disorders.

**Results:**

From 2006 onward, with no decrease in the number the beds, the number of inpatient days for first-in-life patients with schizophrenia decreased by 29%, their admission rates dropped by 22%, the proportion of short [< 30 days] first in life episodes went up, while the percentage of those whose first in life episode lasted more than one year went down from 2.5% to 0.5%. The parallel results for patients with affective disorders were much less significant.

**Conclusions:**

An increasing percentage of patients with schizophrenia are not admitted to psychiatric wards at all and an increasing percentage of those who are admitted are treated during a shorter episode. The change is probably due to the rehabilitation reform which enabled the structural reform (the reduction in beds) to be implemented effectively.

## Background

During the last half century, psychiatric reforms, intending to integrate the mentally ill into the community [1-4], as well as the development of new neuroleptic drugs [5] reduced significantly the importance of inpatient care for people with severe mental disorders [6,7].

Examination of trend data on inpatient care in Israel from the 1960’s to the1990’s revealed a dramatic reduction in the accumulated length of inpatient stay among patients with schizophrenia until the 1980’s, but no further reduction during the 1990’s [8]. The authors attributed these changes to the use of long acting medication, the increase in the number of outpatient clinics and the commencement of social security benefits. Among patients with affective disorders, the reduction in inpatient length of stay was less prominent and occurred mainly between the years 1960 and 1970, presumably as a result of the introduction of lithium treatment in Israel [8].

Following the trend of deinstitutionalization around the world, the Ministry of Health, together with the Ministry of Finance, launched a ‘structural reform’ of the psychiatric hospitals in 2001. The aim of the reform was to actively reduce the number of psychiatric beds to a specified bed ratio of 0.5/1,000 population, to a targeted inpatient length of stay (a mean of 33 days for acute patients) and to an admission rate that should not be higher than 3.2/1,000 [9], in order to limit inpatient services to patients in the acute phase of their disease [9].

Since all psychiatric inpatient facilities (psychiatric hospitals and psychiatric departments in general hospitals) are budgeted in Israel by the government, the reduction of beds was carried out by shifting some of the budget from inpatient care to the community. The structural reform was enabled by the ‘Rehabilitation of the Mentally Disabled in the Community Law’ [10], which was passed in 2000. This innovative law spurred the transfer of inpatients with chronic conditions to rehabilitation programs in the community by allocating funds to a variety of residential and vocational programs [11]. The rehabilitation reform was designed to advance the deinstitutionalization of chronic patients, i.e. mostly patients with schizophrenia.

Initial analysis of the effects of both reforms showed a dramatic decrease (24.3%) between 2000 and 2004, in the number of patients whose length of stay exceeded one year, but showed only a small decrease in the average length of acute stays for adults from 37.6 days in 2000 to 36.4 days in 2004 [12]. However, because the analysis was restricted to data through 2005, and did not involve separating first-in-life patients from readmitted patients, it may not have detected all the effects of the reforms and may have masked hospitalization patterns of specific diagnostic subgroups.

The rehabilitation reform was tailored specifically to patients with chronic psychiatric disability which is typical mainly of schizophrenic patients. The main objective of the present paper was therefore to examine the impact of the above policy changes on the hospitalization patterns of patients with schizophrenia.

We assumed that the main effect of the rehabilitation reform will be on the length of inpatient stay of patients with schizophrenia and that changes in the system will manifest themselves first, and most prominently, on new patients who were not habituated to the previous patterns of care.

We therefore separated the analysis of first-in-life patients vs. readmitted patients, and tracked the hospitalization patterns of patients with schizophrenia in comparison to those of patients with affective disorders as a useful comparison group. This is because, while the structural reform had the potential to affect both groups, the rehabilitation reform was targeted primarily at the schizophrenia patients.

Specifically we focused on the changes that occurred in the rate of admissions, length of hospitalizations, percentage of first in life episodes lasting more than one year, total inpatient days and tenure in the community after discharge from first-in-life episodes.

## Methods

Data on all psychiatric admissions during the period 1990–2009 were used for the analysis. A follow-up of the hospitalizations of the individual patients was done for the same period. The data (without any identifying information of the disabled persons) were extracted from the Israel Central Psychiatric Case Register [13]. This database includes demographic variables, diagnosis and dates of all admission to, and discharge from, psychiatric hospitals or from psychiatric departments in general hospitals. Hospitals in Israel have been required by law to report this information to the Central Register since 1950.

### Definition of measures

*First-in-life admission rates*: This measure included all first admissions of individuals who were born in Israel or who immigrated to Israel before the age of 18 and whose diagnosis at discharge from the first admission was schizophrenia, schizotypal or delusional disorders [F20-F29] or affective disorder [F30-F39] according to the ICD-10 [14]. Since more than 70% of the patients in the [F20-F29] category were diagnosed as suffering from ‘schizophrenia’, we refer to this group in the study as ‘patients with schizophrenia’.

Rates were expressed as a ratio between the number of first admissions each year and the size of the population of Israel aged 15 and above, in that year.

*Total admission rates*: This measure included all admissions of individuals whose diagnosis at discharge was schizophrenia, schizotypal or delusional disorders [F20-F29] or affective disorder [F30-F39] according to the ICD-10 [14]. Rates were expressed as a ratio between the number of all admissions each year and the size of the population of Israel aged 15 and above, in that year.

*Length of first-in-life episodes*: The number of days from admission to discharge of the first-in-life episodes was divided into four groups: 1–30 days, 31–60 days, 61–90 days and 91 days and more.

*Percentage of patients whose first-in-life episodes lasted more than one year*: The number of first-in-life patients with schizophrenia or affective disorders whose length of episode was longer than 365 days, divided by the total number of first–in-life patients with schizophrenia or affective disorders in that year.

*Percent of patients who remained in the community for at least 3 years after discharge from a first-in-life admission which lasted less than 365 days*: Length of stay in the community was calculated from the day of discharge to the readmission day. For patients who were not readmitted within the follow up period, length in the community was calculated from the day of discharge to the end of the follow up period. To enable comparisons between years, the analysis included only those patients who had a follow up period which was at least 3 years long. Thus, the measure presents the percentage of those who were not admitted within the 3 years of follow up out of all those who had a follow up period of at least 3 years.

### Statistical analysis

Analyses were conducted using STATISTICA V.6 [15]. The data were summarized by year using cross-tabulations and frequency distributions.

## Results

### Beds and inpatient days

**Figure 1:**
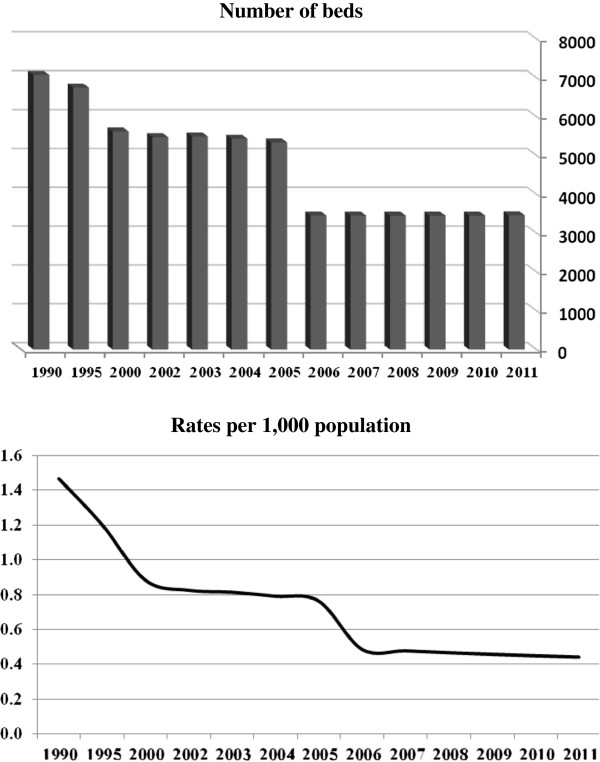
Number and rates of psychiatric beds per population.

Figure 1 shows that in the 10 years between 1990 and 2000, there was a decrease of 20% in the number of beds for psychiatric hospitalization from 7,074 to 5,619. The implementation of the structural reform accelerated the rate of decrease, so that between 2000 and 2006, 40% of the beds were removed and at the end of 2006 there were only 3,453 psychiatric beds. Since then there has not been any further decrease in the number of psychiatric beds, but an increase in population size lowered the bed ratio per population from 0.49 per 1,000 population in 2006 to 0.45 per 1,000 population in 2010.

**Figure 2:**
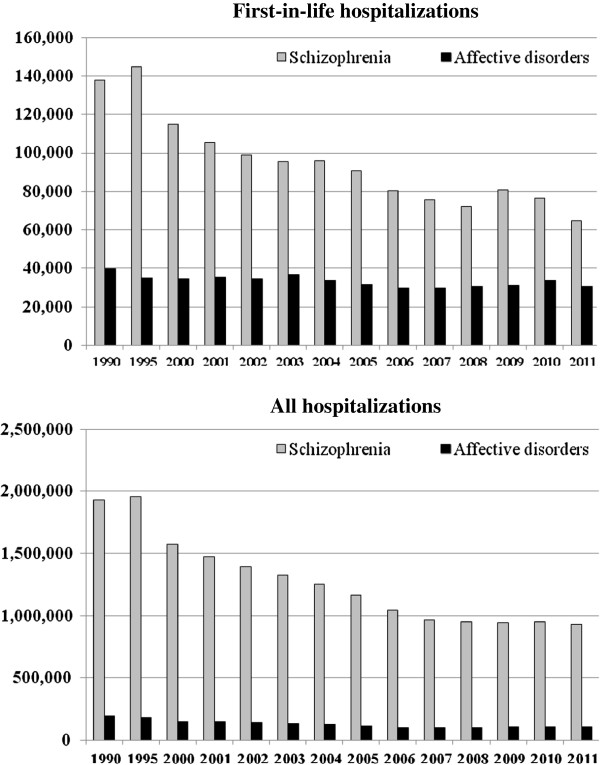
Inpatients days by diagnosis and year.

Figure 2 shows the differential change in the number of inpatient days following the reduction in psychiatric beds during the period between 1990 and 2011 for first-in-life patients and for all patients, in the two diagnostic groups: patients with schizophrenia and patients with affective disorders. The number of inpatient days of first-in-life patients with schizophrenia went down from 137,765 days in 1990 to 114,925 in 2000 - a decrease of 17% - and then continued to drop between 2000 and 2006. After 2006 there was an additional decrease to 64,627 by 2011.

The same pattern of decrease can be seen also in the number of inpatient days for all patients with schizophrenia. A drop of 25% was observed in 2000 compared to 1990, but the number dropped by a further 50% by 2011. It is important to note that the decrease in inpatients days continued between 2006 and 2011, even though there was no decrease in the number of beds during that period. The decrease in the number of inpatient days was less remarkable for patients with affective disorders. Among first-in-life patients, there was a slow decrease over the years which resulted in a 22% decrease between 1990 and 2011.

### Rates of admission

**Figure 3:**
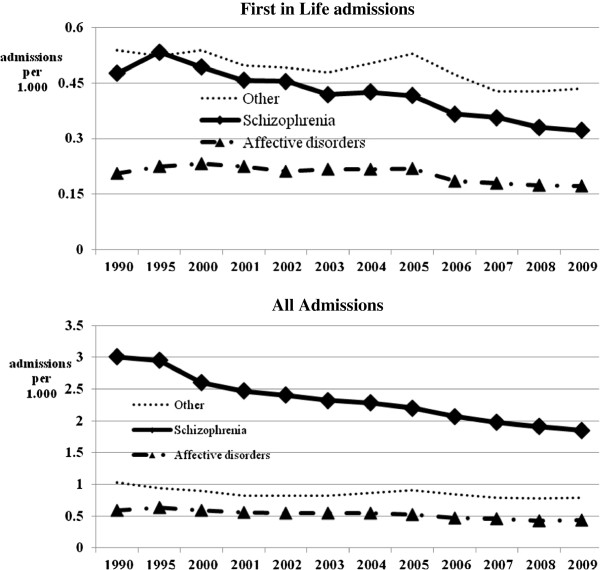
Rates of admissions per 1000 population among 15 years old and above.

Figure 3 shows the rates of admission for the 15 and above age group of the population, for first-in-life patients and for all patients in the two diagnostic groups.

Admission rates of first*-*in-life patients with schizophrenia declined from a peak of 0.53/1,000 in 1995 to 0.49 in 2000 and then to 0.37 in 2006. Between 2006 and 2009 the rate declined by an additional 14% to 0.32/1,000. The rate of admission of first-in-life patients with affective disorders declined relatively less, from 0.23/1,000 in 2000 to 0.17/1,000 in 2009.

Total admission rates of patients with schizophrenia declined from 3.0/1,000 in the nineties to 2.6/1,000 in 2000 and then by another 30% to 1.8/1,000 in 2009, while the parallel rates for patients with affective disorders declined only by about 25% from 2000 till 2009.

### Length of first-in-life episodes

**Figure 4:**
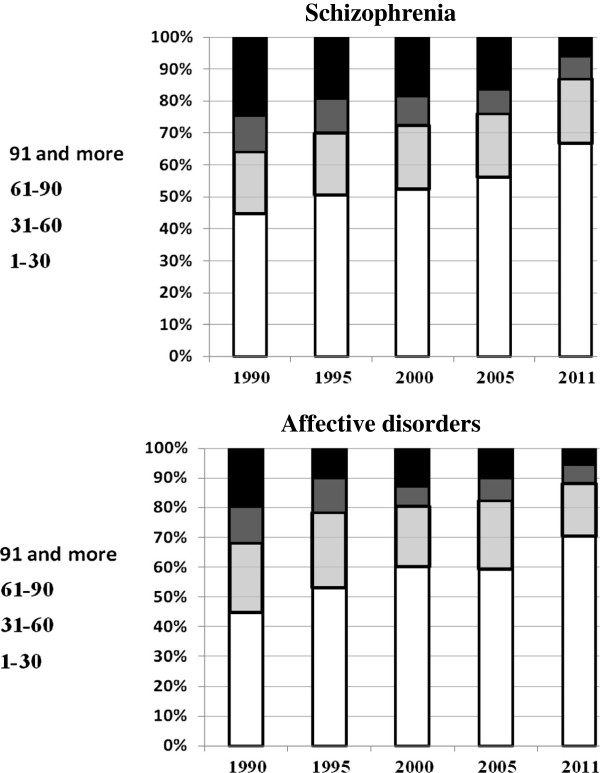
Distribution of first-in-life episodes by length of episodes and year.

Figure 4 shows the distribution of first-in-life episodes by length of episode over the years. It shows that, during the 90’s, about 40% of first-in-life patients with schizophrenia had a first episode of 30 days or less and about 25% of them had a first episode of 90 days or longer.

In 2010, patients with short episodes (i.e., those of 30 days or less) comprised 70% of the entire group and those with the long episodes (i.e., those of 90 days or longer) comprised less than 10% of the group.

A similar pattern appears for patients with affective disorders. The proportion of short episodes went up from about 40% to 70% and the proportion of long episodes went down from about 20% to less than 10% in that period.

Again, the decrease in the percentage of those with long episodes continued at the same rate between 2005 and 2011, even though there was no decrease in the number of beds during that period.

### Proportion of patients whose first-in-life episode lasted more than 1 year

**Figure 5:**
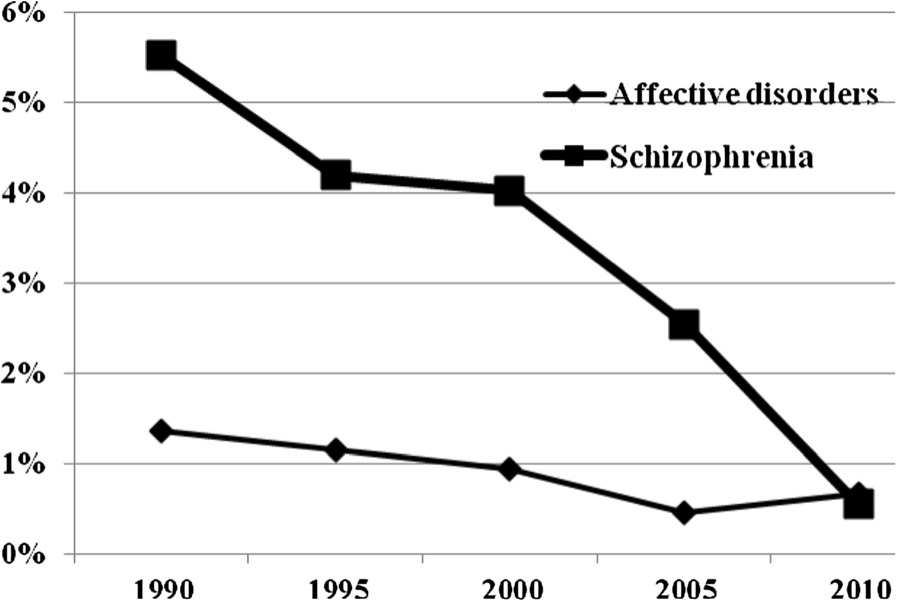
Percentage of patients whose first-in-life episode lasted more than 1 year.

Figure 5 shows the decline in the proportion of patients whose first-in-life episode lasted more than 1 year. In 1990, 5.5% of the first-in-life patients with schizophrenia stayed in the hospital for more than 365 days, in 2000 it was 4%, while in 2010 only 0.5% of the patients needed to stay that long.

### Readmission after first-in-life episode

**Figure 6:**
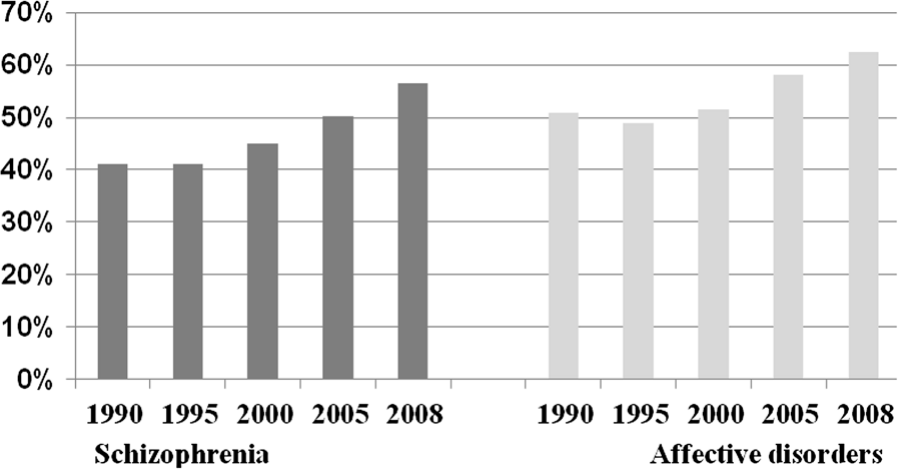
Percent of first-in-life patients who were not readmitted within 3 years from discharge.

Figure 6 shows the proportion of patients who stayed in the community for at least 3 years after their first admission. The proportion increased from 40% in 1990 to 50% in 2005 and then continued to increase to 56% in 2008 for patients with schizophrenia. A similar trend was found for patients with affective disorders.

## Discussion

The main objective of the present paper was to assess whether the reforms implemented by the Ministry of Health during the 2000’s advanced the shift of psychiatric patients from inpatient settings to the community.

The results show that during the 2000’s there was a striking decrease in the number of psychiatric beds to a rate of 0.45 per 1,000 population. The decrease in the number of beds, to one of the lowest among the OECD countries [16], paralleled a reduction in the number of inpatient days, mainly among patients with schizophrenia.

One might argue that the reduced number of inpatient days is a direct result of the shortage of beds and does not necessarily show that there was a reduced demand for inpatient days. However, this ceiling effect argument cannot be sufficient in our case because the reduction in the number of inpatient days for patient with schizophrenia continued between 2006 and 2011 even though the number of beds did not change during these years. A ceiling effect would have forced patients to be discharged earlier, but the total number of inpatients days would have remained the same.

A more thorough examination suggests that the changes in the pattern of use described above are linked to a large extent to the two inter-related reforms. The comparison between patients with schizophrenia and patients with affective disorder shows that the biggest change after the reform was observed among those who were the primary target of the reforms, (and particularly the rehabilitation reform) patients with schizophrenia.

The rate of first-in-life admissions of patients with schizophrenia, who are the main group among the first-in-life admissions, went up by 4% from 1990 to 2000 but then went down by an impressive 35% in the period until 2009.

Shortage of beds does not explain results like these as no parallel decrease was observed among the patients with affective disorders. The decrease in the admission rate of patients with schizophrenia also cannot be attributed to the pharmacological effect of the new generation of antipsychotic drugs since these were already in use in the early nineties [17]. It seems plausible that this marked decrease in admission rates of patients with schizophrenia is related to the reform in the rehabilitation services and the new opportunities it offered for keeping these patients in the community.

Moreover, patients with schizophrenia not only had fewer first-in-life admissions but also had a higher percentage of relatively short first-in-life episodes during the 2000s compared to the nineties, with a lower percentage of them remaining hospitalized for more than one year after admission and an increasing percentage of them remaining in the community without readmission for at least 3 years. A parallel result for the early part of the decade was reported by Grinshpoon et.al [18] which showed that patients with schizophrenia who had been hospitalized for 6 months or more had lower readmissions rates in 2000–2001 compared to 1990–1991, while no similar decrease was observed among patients with affective disorders.

Given that the incidence rate of the schizophrenia spectrum is stable around the world at about 0.3 /1,000 [19], these results imply that services in the community such as supported housing or vocational rehabilitation managed to better meet the needs of this new generation of first-in-life patients with schizophrenia so that their need for hospital stay decreased. This suggests that an increasing percentage of the new patients with **s**chizophrenia can possibly be treated in the community and an increasing percentage of those who are admitted can be treated effectively via a shorter, once in a lifetime inpatient episode. These results are in line with a worldwide trend [20-22].

One might claim that the reduction in admissions may have led to a deterioration in the clinical condition of the more severe patients in the community. However, if such a deterioration had taken place, we would have expected to find a substantial increase in the proportion of compulsory admissions among all admissions. In fact, however, that proportion increased only slightly - from 24% in 2000 to 28% in 2010 [Ministry of Health, 2010]. Moreover, there were no major changes in the policies or practices regarding mandatory hospitalization in the last 10 years that might have confounded the use of this indicator.

In summary, changes in the care of psychiatric patients following the rehabilitation reform and the structural reform are likely to account for the sharp decrease in inpatient stays among new psychiatric patients. The generator of the change was probably the rehabilitation reform which created new types of services to accommodate the needs of the more chronic patients and enabled the structural reform to be implemented.

### Limitations

The main limitation of the study stems from the fact that the reforms were implemented simultaneously nationwide, and therefore there was no ideal control group for the trends in hospital use. Yet, the comparison between patients with schizophrenia (for whom the rehabilitation reform was most relevant) and patients with affective disorders does provide a partial control and thus helps tease out the effects of the reforms.

The study does not examine other social or clinical forces that might have contributed to a decline in admission rates for patients with schizophrenia, including those that might have involved a shift from inpatient to community care. Such forces might include a possible increase in the volume of community mental health services beside the rehabilitation network, or possible changes in attitudes to psychiatric patients. We doubt however that changes in these areas could have caused the changes that we observed.

Finally, this study does not examine how the changes in treatment patterns may have affected patients’ health. It is recommended that a follow up study on a cohort of patients with serious mental disorders be added to the evaluation of the rehabilitation reform, so that the clinical and functional status of these patients can be closely monitored.

## Conclusions

The relative success in the implementation of the two reforms suggests that the personnel in mental health within and outside the hospitals, the medical technology and the public were ready for these reforms. The transfer of the responsibility for mental health to the health plans after the expected insurance reform for mental health, which is expected to promote parity and integration between mental and physical health services, might even reinforce the observed trends by integrating mental and physical health.

The number of psychiatric hospitalizations is likely to continue to rise for the mere fact that the population in Israel increases yearly by almost 2%. It is therefore not recommended to reduce the number of beds further.

## Competing interests

The authors declare that they have no competing interests.

## Authors’ contributions

Both authors were involved in all aspects of the study. Both authors read and approved the final manuscript.

## Authors’ information

Daphna Levinson is the head of the research and planning unit in the Mental Health Services Section of the Ministry of Health. Yaacov Lerner is the former director of the Jerusalem Mental Health Center. He is a specialist in mental health service research. He is presently the director of the Falk Institute for Mental Health Research, Jerusalem, Israel.
